# How aesthetic education enhances psychological resilience: the mediating role of emotion regulation

**DOI:** 10.3389/fpsyg.2026.1802363

**Published:** 2026-04-15

**Authors:** Qingqin Zhu, Yu Dong, Hanfeng Chen

**Affiliations:** 1 School of Creative Studies, Yiwu Industrial and Commercial College, Jinhua, China; 2 School of Computer Science and Technology, East China Normal University, Shanghai, China

**Keywords:** aesthetic education, cognitive reappraisal, emotion regulation, expressive suppression, psychological resilience

## Abstract

**Introduction:**

Aesthetic education is widely assumed to foster students' emotional and psychological development; however, empirical evidence from vocational education contexts remains limited. Drawing on emotion regulation theory, this study examines whether participation in aesthetic education enhances vocational students' psychological resilience through cognitive reappraisal and expressive suppression.

**Methods:**

Survey data were collected from 412 vocational students in China. Aesthetic education participation, cognitive reappraisal, expressive suppression, and psychological resilience were measured using self-report scales, and the data were analyzed using structural equation modeling.

**Results:**

The results indicate that greater participation in aesthetic education significantly predicts higher levels of cognitive reappraisal and lower levels of expressive suppression, which in turn are significantly associated with enhanced psychological resilience. The indirect effects through cognitive reappraisal and expressive suppression were both significant, supporting the proposed mediating role of emotion regulation.

**Discussion:**

These findings suggest that aesthetic education functions as a non-threatening and experience-based psychological intervention by facilitating adaptive emotion regulation processes. The study highlights the psychological value of aesthetic education in vocational settings and offers practical implications for curriculum design and students' emotional development in skill-oriented educational contexts. Given the cross-sectional design, the findings should be interpreted as evidence of associations rather than causal effects.

## 1 Introduction

Aesthetic education has long been regarded as an important component of holistic education (D'olimpio, 2022), extending beyond the cultivation of artistic skills to encompass sensory experience, emotional engagement, and reflective meaning-making. Through participation in arts-based and creative activities, students are encouraged to explore emotions, interpret experiences, and engage with ambiguity in non-instrumental learning contexts. As such, aesthetic education is increasingly viewed as a psychologically relevant educational environment in which emotional experiences are actively generated, expressed, and regulated, rather than merely a channel for artistic training (Li and Sun, 2023).

While previous research has demonstrated that aesthetic education is associated with positive psychological outcomes such as subjective wellbeing and life satisfaction (Ye et al., 2025; Minmin and Mo, 2025), these indicators primarily reflect individuals' general evaluations of emotional states and quality of life. In many existing educational models, positive psychological development is primarily operationalized through wellbeing or general psychological adjustment indicators. However, these outcomes do not directly capture students' stress-adaptive capacity under adversity. Psychological resilience, defined as the ability to maintain or regain psychological functioning under challenging conditions, represents a distinct and functionally oriented outcome that cannot be fully captured by general wellbeing indicators (Troy et al., 2023; Bonanno et al., 2023). From this perspective, investigating psychological resilience allows for a more process-oriented understanding of how educational experiences contribute to students' capacity to cope with stress, recover from setbacks, and sustain engagement in demanding learning environments.

This distinction is particularly salient in vocational education contexts. Compared with academically oriented university pathways, vocational education places stronger emphasis on skill performance, employability, and direct labor-market transition. Students in vocational institutions often encounter heightened performance pressure, frequent external evaluation, and uncertainty regarding future career trajectories (Wang and Wang, 2023; Zeng et al., 2022). These contextual characteristics may increase students' vulnerability to emotional stress while simultaneously limiting opportunities for open emotional expression, thereby placing greater demands on their emotion regulation capacities (Zhou et al., 2023). Despite these challenges, existing research on vocational education has predominantly focused on technical competencies, career readiness, and employment outcomes (Lindsay et al., 2024; Scandurra et al., 2024), with relatively limited attention paid to the psychological mechanisms through which educational experiences support students' adaptive functioning under stress.

Emotion regulation theory provides a well-established psychological framework for explaining how individuals manage emotional experiences in response to situational demands (Gross, 1999). Emotion regulation refers to the processes through which individuals monitor, evaluate, and modify the intensity, duration, or expression of emotions. Among various regulation strategies, cognitive reappraisal and expressive suppression have been identified as two core and theoretically distinct approaches (Brockman et al., 2017). Cognitive reappraisal involves reinterpretation of emotional situations and is generally associated with adaptive psychological outcomes, whereas expressive suppression involves suppressing emotional expression and is often linked to emotional costs and maladaptive functioning. Because emotion regulation strategies operate at the level of emotional processing rather than outcome evaluation, they offer a proximal mechanism through which educational environments may shape students' longer-term adaptive capacities, including psychological resilience.

Aesthetic education may play a particularly important role in shaping students' emotion regulation strategies (Hepburn, 2010). Aesthetic learning environments emphasize emotional awareness, creative exploration, and reflective engagement, providing students with opportunities to experience and reinterpret emotions in a relatively non-threatening contexts (Møller-Skau, 2024). Through engagement in aesthetic activities, students may practice reappraising emotional experiences and expressing feelings in constructive ways, thereby strengthening adaptive emotion regulation capacities.

Building on emotion regulation theory (Gross, 1999) and contemporary affect-regulation accounts of resilience (Troy et al., 2023), the present study examines whether participation in aesthetic education enhances vocational students' psychological resilience through emotion regulation processes. Specifically, this study proposes that aesthetic education participation is positively associated with cognitive reappraisal and negatively associated with expressive suppression, which in turn contribute to higher levels of psychological resilience. By adopting a mechanism-oriented perspective, this study moves beyond descriptive associations between aesthetic education and positive psychological outcomes, focusing instead on how educational experiences are transformed into adaptive psychological capacities in vocational education contexts.

This study contributes to the literature in three ways. First, it extends aesthetic education research by integrating emotion regulation theory and focusing on psychological resilience as a stress-adaptive outcome rather than general wellbeing. Second, it elucidates the proximal emotional mechanisms through which aesthetic education exerts its psychological effects, highlighting the distinct roles of cognitive reappraisal and expressive suppression. Third, by situating the analysis within vocational education, the study addresses a population and educational context that has been underrepresented in educational psychology research.

## 2 Literature review and development of hypotheses

### 2.1 Aesthetic education and students' psychological development

Growing research evidence in the field of educational psychology suggests a significant positive correlation between aesthetic education and students' psychological development (Smith, 1970; Zhang, 2021). Broadly speaking, aesthetic education refers to educational activities centered on artistic participation, sensory experience, and creative expression. Existing research has found that aesthetic education is closely related to various emotional and psychological outcomes, including emotional awareness, subjective wellbeing, and mental health (Burt, 1967). Recent empirical studies suggest that participation in art-related educational activities activities can provide structured opportunities for emotional experience, expression, and reflection, and is associated with better emotional functioning and psychological adjustment in student populations (Guo, 2024; Zhang and Zhao, 2024).

Specifically, aesthetic education and aesthetic education participation are significantly associated with lower levels of emotional distress and higher levels of subjective wellbeing (D'Olimpio, 2022). For example, large-scale empirical studies and systematic reviews consistently indicate a positive correlation between aesthetic participation and students' emotional wellbeing, stress relief, and psychological resilience, and this trend is evident across different age groups and educational stages (Nutbrown, 2013; Lawton et al., 2019). These findings suggest that aesthetic education provides a meaningful context for students' emotional development.

In addition to emotional outcomes, recent research has increasingly emphasized the role of aesthetic education in promoting broader psychological capabilities. Art-based learning environments are typically characterized by openness, exploration, and tolerance for ambiguity, which contribute to fostering students' psychological flexibility and adaptive coping abilities (See and Kokotsaki, 2016). Empirical evidence suggests that students receiving art or creative education experiences tend to exhibit higher levels of emotional engagement, reflective thinking, and self-awareness—all crucial foundations for healthy psychological development (Rezaei, 2025).

However, despite the increasing body of research, existing literature primarily focuses on general and higher education settings, with relatively insufficient attention paid to vocational education. Most studies view aesthetic education as a supplement to academic curricula, emphasizing its positive impact on students' overall wellbeing while delving less into its specific psychological mechanisms. Therefore, although a growing consensus has emerged that aesthetic education has a positive impact on students' psychological development, how it operates through specific emotional processes, especially among vocational students facing unique developmental challenges, remains to be further explored.

### 2.2 The concept of psychological resilience and its role in educational research

Psychological resilience refers to an individual's ability to effectively adapt and recover from adversity, stress, or trauma. It is a dynamic process involving the interaction between individual traits and external resources that facilitate adaptive functioning in the face of challenges (Masten, 2025). Rather than a fixed trait, resilience is cultivated and strengthened through various experiences, including educational and social contexts (Gong et al., 2023). In recent years, it has become a central topic in psychological and educational research due to its relevance for students' coping with academic, emotional, and social challenges.

In educational contexts, psychological resilience is closely associated with academic success and overall wellbeing (Wu et al., 2020). Psychologically resilient students tend to manage stress more effectively, cope with setbacks, and sustain motivation in the face of difficulties (Ononye et al., 2022; García-Martínez et al., 2022). Resilience is also related to emotion regulation, self-efficacy, and adaptive coping strategies (Romo et al., 2025), underscoring its importance for students' adaptation across academic and social domains.

Recent research emphasizes the need to cultivate resilience within educational frameworks, particularly in vocational education settings where students may encounter career uncertainty and skills-related pressures (Yüksel and Açıkel, 2024). Vocational students often face elevated stress levels due to the direct transition from education to employment, and their capacity to navigate such challenges is closely linked to psychological resilience (Many and Mercier, 2025). Thus, strengthening resilience is critical not only for mental health but also for academic performance and career readiness.

In educational settings, resilience development is supported by protective factors such as supportive relationships, positive school climates, and opportunities for skills development and reflection (Alvord and Grados, 2005). Interventions targeting affective and social competencies—including adaptive emotion regulation, problem-solving, and goal setting—have been shown to enhance students' resilience (Cherian, 2024; Mestre et al., 2017). Collectively, these findings indicate that psychological resilience is a malleable construct that can be fostered through structured educational experiences.

### 2.3 Emotion regulation theoretical framework and core strategies

Emotion regulation refers to a series of processes by which individuals influence the type, intensity, and duration of their emotional experiences (Gross, 2008). It is a core concept for understanding how individuals cope with emotional responses in various situations, particularly those involving stress, uncertainty, or adversity (Gross, 2015).

Emotion regulation theory is primarily grounded in Gross's process model (Gross, 2002), while also being understood as a context-sensitive and flexible regulatory system across different domains (Bonanno et al., 2024; Isaacowitz and English, 2024), including educational settings (Aldrup et al., 2024; Martínez-Priego et al., 2024). In the present study, emotion regulation is operationalized through two ERQ-based strategy dimensions—cognitive reappraisal and expressive suppression—as theoretically distinct representatives within this framework. This operationalization serves as a parsimonious approach and does not imply that these two strategies exhaust the broader construct of emotion regulation.

Cognitive reappraisal refers to an individual altering the emotional impact of a situation by reinterpreting its meaning (Clark, 2022; McRae et al., 2012). Because this strategy operates in the early stages of emotion generation, it is often associated with more adaptive psychological outcomes, such as greater emotional flexibility, more effective stress coping, and more positive psychological functioning (Buhle et al., 2014).

In contrast, expressive suppression refers to the suppression of overt emotional expression after emotions have already arisen (Gross and Cassidy, 2019). Because it operates in the later stages of emotional responses, expressive suppression is often accompanied by higher psychological and physiological costs (Caramanica et al., 2023).

Recent research has highlighted the importance of emotion regulation in both clinical and non-clinical populations (Lincoln et al., 2022; Miu et al., 2022; Larsson et al., 2025). In educational settings, emotion regulation plays a vital role in students' coping with academic stress and interpersonal challenges. Research indicates that emotion regulation is not merely a stable individual difference, but a malleable psychological process influenced by context and educational experiences (Jacobs and Gross, 2014). Learning environments that encourage emotional awareness and reflection are more conducive to the formation of adaptive regulation strategies such as cognitive reappraisal (Santos Alves Peixoto et al., 2022), whereas environments that discourage emotional expression may reinforce reliance on expressive suppression.

From this perspective, aesthetic education, characterized by emotional experience and creative expression, may shape students' emotion regulation styles. Through activities such as music, art, and performance, students are encouraged to explore and reflect on their emotions. These experiences may provide opportunities to practice cognitive reappraisal and constructive emotional expression in supportive contexts, potentially creating conditions conducive to adaptive emotion regulation and subsequent psychological adjustment (Peters et al., 2024).

Accordingly, the associations proposed in this study are conceptualized at the level of relational patterns rather than deterministic causal effects, consistent with the cross-sectional design.

Based on the above theoretical analysis, this study proposes the following hypotheses:

H1: Aesthetic education participation is significantly positively correlated with cognitive reappraisal.

H2: Aesthetic education participation is significantly negatively correlated with expressive suppression.

### 2.4 The relationship between emotion regulation and psychological resilience

Psychological resilience reflects an individual's ability to adapt positively to stress and adversity (Fletcher and Sarkar, 2013). In educational contexts, emotion regulation, by influencing an individual's assessment and coping processes of stressful events, is considered an important proximal mechanism for resilience formation (Şahin and Hepsöğütlü, 2018).

Cognitive reappraisal is widely recognized as an adaptive emotion regulation strategy associated with higher psychological resilience (McRae, 2016). By reconstructing the meaning of stressful or adversarial events, individuals who frequently use cognitive reappraisal are more likely to maintain emotional balance, view challenges as manageable situations, and effectively mobilize coping resources (Wolgast et al., 2011). Therefore, cognitive reappraisal is generally associated with higher levels of psychological resilience and more effective stress recovery.

Conversely, expressive suppression is generally associated with poorer resilience outcomes. Suppressing emotional expression does not reduce an individual's internal negative emotional experience; instead, it may increase physiological arousal levels and emotional load. Individuals who rely on expressive suppression long-term often struggle to effectively process stressful experiences, thus weakening their ability to recover from adversity and maintain psychological resilience (Qu et al., 2024).

Based on the above theories and research evidence, it is reasonable to infer a directional difference between different emotion regulation strategies and students' psychological resilience. Therefore, this study proposes the following hypothesis:

H3: Cognitive reappraisal is significantly positively correlated with psychological resilience.

H4: Expressive suppression is significantly negatively correlated with psychological resilience.

### 2.5 The mediating role of emotion regulation

The preceding sections indicate a close relationship between aesthetic education participation and emotion regulation, as well as between emotion regulation and psychological resilience. Rather than operating through simple direct effects, educational experiences typically influence long-term psychological outcomes by shaping proximal psychological mechanisms. In this context, emotion regulation represents a theoretically grounded mediating process linking aesthetic education participation to psychological resilience. While emotion regulation is a broader construct encompassing multiple strategies, the present study focuses specifically on cognitive reappraisal and expressive suppression as representative dimensions.

Aesthetic education is characterized by emotionally engaging and reflective learning processes (Best, 1978). In art activities, students are encouraged to attend to their emotional experiences and reinterpret emotional situations, processes closely aligned with cognitive reappraisal. At the same time, aesthetic education often legitimizes emotional expression and weakens normative pressures toward emotional suppression, potentially reducing students' reliance on expressive suppression as a regulatory strategy (Zheng et al., 2024). Accordingly, aesthetic education participation may shape students' emotion regulation tendencies by promoting adaptive strategies and reducing maladaptive patterns.

Emotion regulation strategies are closely implicated in psychological resilience. Cognitive reappraisal helps individuals reinterpret stressful events in more constructive ways, reducing emotional intensity and supporting adaptive coping (Álvarez-Huerta et al., 2025). In contrast, expressive suppression may undermine psychological resilience by increasing emotional load and constraining emotional processing (Rea-Sandin et al., 2021). Thus, emotion regulation constitutes a proximal mechanism connecting educational experience and resilience.

**Figure 1:**
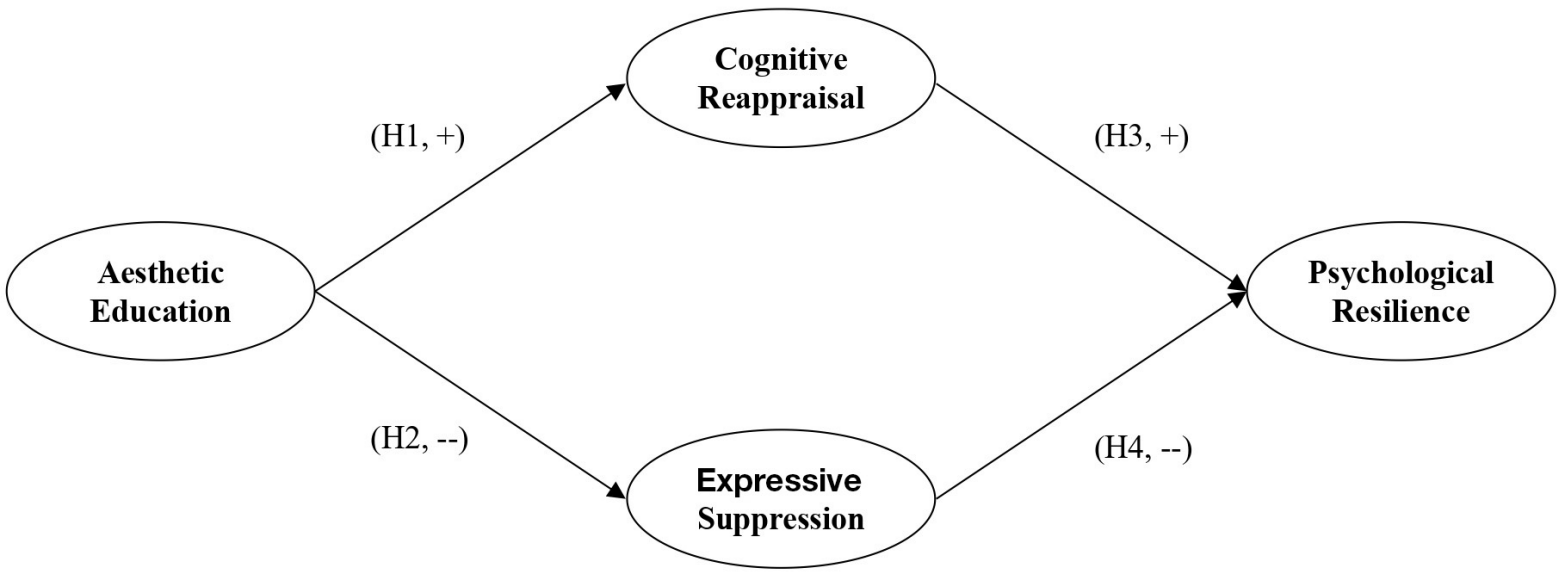
The conceptual model.

In summary, emotion regulation can be conceptualized as the mediating pathway through which aesthetic education participation is associated with psychological resilience. Specifically, increased cognitive reappraisal and decreased expressive suppression represent two complementary mediating processes between aesthetic education participation and psychological resilience (Figure 1).

Based on this integrative framework, this study proposes the following mediation hypotheses:

H5a: Cognitive reappraisal mediates the relationship between aesthetic education participation and psychological resilience.

H5b: Expressive suppression mediates the relationship between aesthetic education participation and psychological resilience; that is, aesthetic education participation is indirectly associated with higher psychological resilience by reducing expressive suppression.

## 3 Methodology

### 3.1 Participants and procedure

Participants were recruited from four comprehensive vocational colleges located in Zhejiang and Shanghai. Convenience sampling was adopted due to institutional access constraints and the feasibility of administering an online survey during the semester. Students were invited to participate via a standardized online questionnaire link disseminated through class coordinators and student affairs offices. All participation was voluntary, with no course credit or material incentives provided.

Before completing the questionnaire, participants were clearly informed of the research purpose and that their answers would be used solely for academic research. All participants provided informed consent electronically before accessing the questionnaire. This study adhered to ethical guidelines for research involving human participants and has undergone ethical review by relevant institutions.

The questionnaire was distributed through a standardized online survey platform. Participants completed the questionnaire individually, with a completion time of approximately 8–10 min. The questionnaire included content on aesthetic education participation, emotion regulation strategies (cognitive reappraisal and expressive suppression), psychological resilience, and demographic information. To reduce the potential impact of common methodological bias, measurement items for different constructs were placed in different sections of the questionnaire. To ensure participants could accurately understand the questionnaire content and provide effective responses, all scales in this study were distributed in Chinese. For scales initially developed in English, this study used existing Chinese versions or appropriately revised existing translations to ensure clarity and comprehensibility. For scales originally developed in Chinese, the existing versions were used directly.

A total of 500 questionnaires were collected. After removing incomplete questionnaires or those with obvious invalid response patterns, *N* = 412 valid questionnaires were obtained for subsequent analysis. Table 1 described the demographic characteristics of the participants. Participants were aged 17–20 years (M = 18.51, SD = 1.13), and the sample came from various professional backgrounds, including engineering/technology, service, art and design, management/business, and information technology.

**Table 1:** Descriptive characteristics of the sample.

Characteristic	Number (percent)
Gender	
Male	200 (48.6%)
Female	212 (51.4%)
Year in college	
First year	150 (36.4%)
Second year	142 (34.4%)
Third year	114 (27.7%)
Other/prefer not to answer	6 (1.5%)
Field of study	
Engineering and technical trades	94 (22.8%)
Hospitality/tourism/health services	92 (22.3%)
Arts and design	56 (13.6%)
Business and management	96 (23.3%)
Information technology	72 (17.5%)
Other/prefer not to answer	2 (0.5%)

### 3.2 Measures

#### 3.2.1 Aesthetic education participation

Aesthetic education participation was measured using a self-developed scale to assess students' subjective engagement with aesthetic education courses or activities during their school years. Unlike objective participation frequency, this scale focuses on students' emotional investment, reflective engagement, and subjective perception of the value of aesthetic education activities, consistent with the theoretical focus of this study. Based on this construct definition, the AEP scale was self-developed following a standard content-validity-oriented procedure. Item generation was guided by mapping content onto three domains: (a) participation exposure, (b) emotional engagement, and (c) reflective/meaning-making experience. Items were reviewed by experts to ensure clarity and representativeness. The full item list and response anchors for the self-developed AEP scale are provided in Supplementary material to enhance transparency and reproducibility.

The scale contains five items covering the frequency of art education participation, emotional investment, reflective experience, and perceived relevance (e.g., “During school, I regularly participate in aesthetic education-related courses or activities”). All items were rated using a 5-point Likert scale (1 = strongly disagree, 5 = strongly agree), with higher scores indicating higher perceived levels of aesthetic education participation. In this study, the scale demonstrated acceptable internal consistency reliability (Cronbach's α = 0.87).

#### 3.2.2 Emotion regulation

Emotion regulation was measured using the Emotion Regulation Questionnaire (ERQ) developed by Gross and John (2003). This scale assesses two different emotion regulation strategies: cognitive reappraisal and expressive suppression.

The cognitive reappraisal subscale contains 6 items and measures an individual's tendency to regulate emotional responses by reinterpreting emotional situations (e.g., “I control my emotions by changing my perception of things”). The expressive suppression sub-scale contains 4 items and measures an individual's tendency to suppress overt emotional expression (e.g., “I control my emotions by not expressing them”). All items were rated using a 5-point Likert scale (1 = strongly disagree, 5 = strongly agree), with higher scores indicating higher frequency of use of the emotion regulation strategy.

In this study, both the cognitive reappraisal subscale and the expressive suppression sub-scale demonstrated good internal consistency reliability (Cronbach's α = 0.84 for cognitive reappraisal; Cronbach's α = 0.81 for expressive suppression).

#### 3.2.3 Psychological resilience

Psychological resilience was measured using the Connor-Davidson Psychological Resilience Inventory Short Version (CD-RISC-10) (Connor and Davidson, 2003). This scale assesses an individual's ability to adapt and recover positively in the face of stress and adversity. Sample items included “I adapt well to change” and “I usually recover quickly after experiencing setbacks.”

All items were rated using a 5-point Likert scale (1 = strongly disagree, 5 = strongly agree), with higher scores indicating higher levels of psychological resilience. In this study, the scale demonstrated good internal consistency reliability (Cronbach's α = 0.91).

### 3.3 Data analysis

Before hypothesis testing, the data were cleaned and quality checked. Questionnaires with incomplete responses or obvious invalid response patterns (such as identical responses) were excluded and not included in subsequent analysis. Descriptive statistics and bivariate correlation coefficients for each research variable were then calculated. Given that this study used the Likert scale, the distribution of the observed variables was tested. Skewness and kurtosis values for all observed items ranged from −0.37 to 0.16 and from −0.80 to −0.25, respectively, which fall within commonly accepted thresholds (|skewness| < 2; |kurtosis| < 7), indicating no serious deviations from normality. In addition, no extreme outliers were identified based on descriptive inspection. These results support the appropriateness of using maximum likelihood estimation in subsequent CFA and SEM analyses.

Confirmatory factor analysis (CFA) was then used to test the measurement model to assess the construct validity of the research variables. The constructed measurement model contained four latent variables: aesthetic education participation, cognitive reappraisal, expressive suppression, and psychological resilience. Each item was loaded only onto its corresponding latent variable. No items were removed from the final CFA; all indicators were retained as specified a priori. Model fit was assessed using the Comparative Fit Index (CFI), the Tucker–Lewis Index (TLI), and the Root Mean Square Error of Approximation (RMSEA). According to established criteria, CFI and TLI values greater than 0.90 and RMSEA values less than 0.08 indicate acceptable model fit. Given the relatively simple and well-specified measurement structure, high incremental fit indices (e.g., CFI/TLI approaching 1.00) may occur when factor loadings are consistently strong and no improper solutions are present. Furthermore, the discriminant validity among latent variables was further examined by comparing the hypothetical model with several more simplified competing models.

Given that all variables were measured using self-report questionnaires, potential common method bias was examined. Harman's one-factor test was used, loading all items into single latent variables. The results showed that the goodness of fit of the one-factor model was significantly lower than that of the hypothetical measurement model, indicating that common method bias is unlikely to have a significant impact on the research results. In addition, procedural controls were implemented during the questionnaire design phase, such as placing measurement items of different constructs in different parts of the questionnaire, to further reduce the risk of common method bias.

Subsequently, structural equation modeling (SEM) was used to test the research hypotheses. The structural model included direct pathways from aesthetic education participation to cognitive reappraisal and expressive suppression, and direct pathways from cognitive reappraisal and expressive suppression to psychological resilience. To examine the mediating role of emotion regulation strategies, a bootstrap method was used for 5,000 repeated samplings, and bias-corrected 95% confidence intervals were calculated. Indirect effects were considered significant when the confidence interval did not contain zero. The model simultaneously estimated both direct and indirect effects to determine the nature of the mediating effect.

Descriptive statistics and preliminary analyses were conducted using SPSS (version 27.0.1). Confirmatory factor analysis and structural equation modeling were performed using AMOS (version 24). Given that this study used cross-sectional data, the mediation model results should be understood as a theoretically based interpretive framework rather than direct evidence of causality.

## 4 Research results

### 4.1 Descriptive statistics and correlations

Table 2 presents the descriptive statistics and Pearson correlation coefficients for the main variables. Prior to hypothesis testing, the composite scale scores were screened for univariate outliers. Boxplots based on the interquartile range (IQR) criterion indicated no extreme outliers. In addition, standardized scores were examined using a conservative threshold (|*z*| > 3.29), and no cases exceeded this criterion (z-score ranges: AEP = −2.46 to 1.96; CR = −2.43 to 2.00; ES = −2.45 to 2.34; PR = −2.76 to 2.02). Therefore, all *N* = 412 cases were retained for subsequent analyses.

**Table 2:** Descriptive statistics and correlations.

Variable	M	SD	1	2	3	4
1. AEP	3.58	0.72	—			
2. CR	3.72	0.64	0.24^**^	—		
3. ES	2.92	0.78	−0.22^**^	−0.19^**^	—	
4. PR	3.65	0.67	0.22^**^	0.43^**^	−0.30^**^	—

All items were rated on a 5-point Likert scale (1 = strongly disagree, 5 = strongly agree); higher scores indicate higher levels of each construct. *N* = 412.

^**^p < 0.01.

As shown in Table 2, aesthetic education participation is significantly positively correlated with cognitive reappraisal and psychological resilience, and significantly negatively correlated with expressive suppression. Cognitive reappraisal is significantly positively correlated with psychological resilience, while expressive suppression is significantly negatively correlated with psychological resilience. These correlations are consistent with the direction of the research hypotheses and provide preliminary support for the subsequent structural model analysis.

### 4.2 Measurement model

Confirmatory factor analysis (CFA) was conducted to test the measurement model and to assess the structural validity of the research variables. A four-factor model was specified, corresponding to aesthetic education participation, cognitive reappraisal, expressive suppression, and psychological resilience, with each observed indicator loading only onto its respective latent construct.

The results indicated that the hypothesized four-factor model demonstrated an excellent fit to the data ($\chi_{\left(269\right)}^{2}=259.713$, *p* = 0.647, χ^2^/*df* = 0.97, CFI = 1.00, TLI = 1.00, RMSEA = 0.00). Importantly, no correlated residuals were added and no *post-hoc* model modifications were conducted to improve model fit. All standardized factor loadings were statistically significant and exceeded the recommended threshold of 0.50, indicating satisfactory indicator reliability. To further examine discriminant validity and address potential common method bias, alternative competing models were tested. As shown in Table 3, the three-factor model demonstrated a poorer fit ($\chi_{\left(272\right)}^{2}=655.13$, χ^2^/*df* = 2.41, CFI = 0.884, TLI = 0.872, RMSEA = 0.059) compared to the hypothesized four-factor model. The two-factor model showed further deterioration ($\chi_{\left(274\right)}^{2}=1205.14$, χ^2^/*df* = 4.40, CFI = 0.719, TLI = 0.692, RMSEA = 0.091). Importantly, the single-factor model exhibited substantially poor fit ($\chi_{\left(275\right)}^{2}=1635.20$, χ^2^/*df* = 5.95, CFI = 0.589, TLI = 0.552, RMSEA = 0.110), with a significant decrease in model fit relative to the four-factor model ($\Delta \chi_{\left(6\right)}^{2}=1,375.49$, *p* < 0.001, ΔCFI = 0.411). These findings indicate that the measurement structure is best represented by four distinct constructs and that a single common factor cannot adequately explain the covariance among the observed variables. Moreover, chi-square difference tests indicated that all alternative models showed significantly worse fit than the hypothesized four-factor model (all Δχ^2^ tests were statistically significant, *p* < 0.001.).

**Table 3:** Results of confirmatory factor analysis.

Model	χ^2^	*df*	χ^2^/*df*	RMSEA	CFI	TLI
Four-factor model	259.71	269	0.97	0.000	1.00	1.00
Three-factor model (A, B+C, D)	655.13	272	2.41	0.059	0.884	0.872
Two-factor model (A+B+C, D)	1205.14	274	4.40	0.091	0.719	0.692
One-factor model (A+B+C+D)	1635.20	275	5.95	0.110	0.589	0.552

A, Aesthetic education participation (AEP); B, Cognitive reappraisal (CR); C, Expressive suppression (ES); D, Psychological resilience (PR).

Convergent validity of the measurement model was further examined using composite reliability and average variance extracted. As shown in Table 4, the composite reliability values for all constructs exceeded the recommended criterion of 0.70, indicating satisfactory internal consistency. The average variance extracted values for aesthetic education participation, cognitive reappraisal, and expressive suppression exceeded the commonly recommended threshold of 0.50. Although the average variance extracted value for psychological resilience (0.480) was slightly below the conventional 0.50 guideline, its composite reliability was high (0.902), and all standardized factor loadings were statistically significant and above 0.50. Following established methodological recommendations, convergent validity can be considered acceptable when composite reliability is adequate and indicator loadings are substantial, even if average variance extracted is marginally below 0.50. Therefore, the overall convergent validity of the measurement model was deemed acceptable.

**Table 4:** Convergent validity of the measurement model.

Construct	Composite reliability	Average variance extracted
AEP	0.883	0.602
CR	0.861	0.508
ES	0.854	0.594
PR	0.902	0.480

Composite reliability values exceeded the recommended threshold of 0.70, indicating satisfactory internal consistency. AVE values exceeded 0.50 for three constructs; although the AVE for psychological resilience was slightly below 0.50, its convergent validity was considered acceptable in light of its high composite reliability and substantial factor loadings.

### 4.3 Structural model and hypothesis testing

Building on the satisfactory measurement model, structural equation modeling (SEM) was conducted to test the proposed hypotheses. In the structural model, aesthetic education participation was specified as an exogenous variable, cognitive reappraisal and expressive suppression as parallel mediators, and psychological resilience as the outcome variable. No demographic covariates were included in the final structural model.

The results indicated that the proposed structural model demonstrated an excellent fit to the data, $\chi_{\left(271\right)}^{2}=268.31$, *p* = 0.535, χ^2^/*df* = 0.99, CFI = 1.00, TLI = 1.00, RMSEA = 0.00 (90% CI [0.00, 0.02]), and RMR = 0.04. All fit indices met or exceeded commonly recommended criteria, suggesting that the structural model adequately represented the observed data.

**Figure 2:**
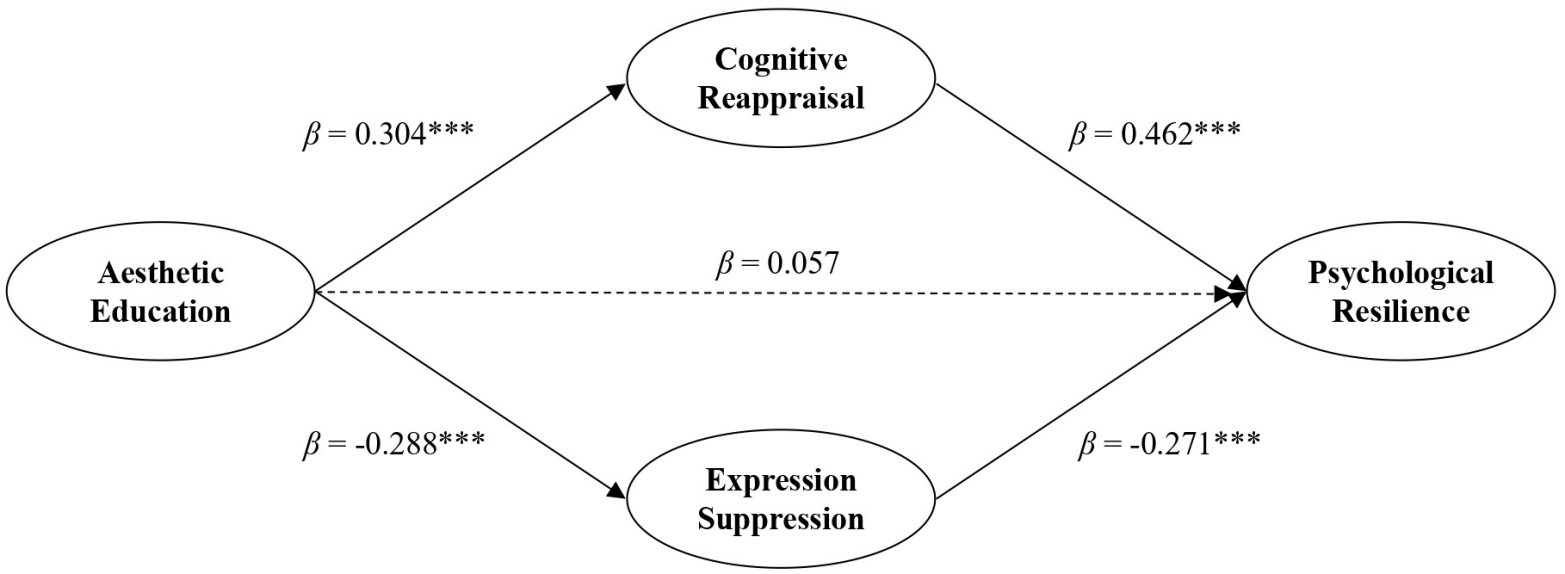
Standardized solution of the SEM. Solid lines represent statistically significant paths; dashed lines represent non-significant paths. ^***^p < 0.001.

At the path level (Figure 2), aesthetic education participation was found to be a significant positive predictor of cognitive reappraisal (β = 0.304, *p* < 0.001), supporting Hypothesis 1. In addition, aesthetic education participation significantly and negatively predicted expressive suppression (β = −0.288, *p* < 0.001), supporting Hypothesis 2. These findings indicate that students who are more engaged in aesthetic education are more likely to adopt adaptive emotion regulation strategies (i.e., cognitive reappraisal) and less likely to rely on maladaptive strategies (i.e., expressive suppression).

Furthermore, cognitive reappraisal was positively associated with psychological resilience (β = 0.462, *p* < 0.001), providing support for Hypothesis 3, whereas expressive suppression was negatively associated with psychological resilience (β = −0.271, *p* < 0.001), supporting Hypothesis 4. This pattern of results suggests that different emotion regulation strategies play opposing roles in shaping students' psychological resilience.

Taken together, the structural model results support the proposed hypotheses and demonstrate that aesthetic education participation influences psychological resilience indirectly through key emotion regulation processes, highlighting the central role of cognitive reappraisal and expressive suppression in this mechanism.

### 4.4 Mediation analysis

Building on the structural path analysis, a mediation analysis was conducted to further examine the role of emotion regulation strategies in the relationship between aesthetic education participation and psychological resilience. Cognitive reappraisal and expressive suppression were simultaneously included as parallel mediators to test their indirect effects.

The results of the structural equation model indicated that aesthetic education participation significantly predicted cognitive reappraisal (β = 0.304, *p* < 0.001) and expressive suppression (β = −0.288, *p* < 0.001). In turn, cognitive reappraisal (β = 0.462, *p* < 0.001) and expressive suppression (β = −0.271, *p* < 0.001) were both significantly associated with psychological resilience. These significant path coefficients provided preliminary evidence for the potential mediating roles of emotion regulation strategies.

To formally test the indirect effects, bootstrapping analyses with 5,000 resamples were performed. As shown in Table 5, the indirect effect of aesthetic education participation on psychological resilience via cognitive reappraisal was statistically significant (β = 0.125, *SE* = 0.029, 95% CI [0.073, 0.186]). In addition, the indirect effect through expressive suppression was also significant (β = 0.068, *SE* = 0.021, 95% CI [0.032, 0.114]).

**Table 5:** Bootstrap estimates of direct, indirect, and total effects.

Effect path	β	*SE*	95% CI
Direct effect (AEP → PR)	0.057	0.054	[−0.045, 0.165]
Indirect effect via CR (AEP → CR → PR)	0.125	0.029	[0.073, 0.186]
Indirect effect via ES (AEP → ES → PR)	0.068	0.021	[0.032, 0.114]
Total indirect effect	0.193	0.037	[0.125, 0.271]
Total effect	0.249	0.057	[0.139, 0.367]

Effects were estimated using structural equation modeling with 5,000 bootstrap resamples. β represents the unstandardized bootstrap mean estimate. CI, confidence interval.

Furthermore, when cognitive reappraisal and expressive suppression were included in the model, the direct effect of aesthetic education participation on psychological resilience was no longer statistically significant, whereas the total indirect effect remained significant (β = 0.193, *SE* = 0.037, 95% CI [0.125, 0.271]). These results indicate that the association between aesthetic education participation and psychological resilience was primarily transmitted through emotion regulation strategies. Notably, the indirect pathway via cognitive reappraisal (β = 0.125) was larger than that via expressive suppression (β = 0.068), indicating a comparatively stronger mediating role of reappraisal. In applied terms, this pattern suggests that aesthetic education may be psychologically beneficial for vocational students' resilience chiefly by fostering adaptive reinterpretation of emotional experiences, while reduced reliance on suppression provides an additional complementary route.

## 5 Discussion

### 5.1 Findings

Grounded in emotion regulation theory, the present study examined the psychological mechanisms through which aesthetic education participation contributes to vocational students' adaptive functioning under stress. Rather than focusing on general positive emotional outcomes, this study conceptualized psychological resilience as a functional capacity that enables students to cope with adversity and maintain psychological stability in demanding educational contexts. The findings indicate that aesthetic education participation is systematically associated with psychological resilience through distinct emotion regulation pathways.

Specifically, aesthetic education participation was found to be positively associated with cognitive reappraisal. This result suggests that engagement in aesthetic education may facilitate students' tendency to reinterpret emotionally challenging situations in more adaptive ways. The experiential and meaning-oriented nature of aesthetic education—characterized by creative exploration, emotional reflection, and symbolic expression—may provide students with opportunities to practice flexible meaning-making, thereby strengthening their use of cognitive reappraisal as an emotion regulation strategy.

In contrast, aesthetic education participation was negatively associated with expressive suppression. This finding indicates that students who are more actively engaged in aesthetic education are less likely to rely on suppressing emotional expression. Aesthetic educational contexts often legitimize emotional experience and expression, creating a relatively non-threatening environment in which emotions can be acknowledged rather than suppressed. Such environments may reduce students' habitual reliance on expressive suppression, a strategy that is often associated with emotional cost and maladaptive psychological outcomes.

Furthermore, cognitive reappraisal and expressive suppression showed opposing associations with psychological resilience. Cognitive reappraisal was positively related to resilience, suggesting that students who more frequently reinterpret emotional experiences are better able to recover from stress and adversity. In contrast, expressive suppression was negatively associated with resilience, indicating that habitual suppression of emotional expression may undermine students' capacity for psychological adaptation. These findings are consistent with prior research emphasizing the functional differences among emotion regulation strategies and their distinct roles in psychological adjustment.

In the structural model, the direct path from aesthetic education participation to psychological resilience was explicitly estimated but was not statistically significant (the bootstrap 95% confidence interval included zero). In contrast, the indirect effects via cognitive reappraisal and expressive suppression were significant. This pattern is consistent with an indirect-only mediation structure within the present model. Therefore, references to a “direct emotional-state pathway” should be understood as a conceptual interpretation rather than as a separate statistical test of unmeasured emotional states.

Taken together, the results support a mechanism-oriented interpretation of the relationship between aesthetic education participation and psychological resilience. The findings are consistent with the interpretation that aesthetic education participation is associated with resilience primarily through shaping how students process, regulate, and respond to emotional experiences under stress, rather than through a residual direct pathway beyond the measured mediators. By influencing proximal emotion regulation processes—specifically increasing cognitive reappraisal and reducing expressive suppression—aesthetic education may gradually contribute to the development of resilience as a functional psychological resource in vocational education settings characterized by performance pressure and emotional constraint.

From a theoretical perspective, the present findings contribute to the literature by specifying a mechanism-based pathway linking aesthetic education participation to resilience through ERQ-based regulation strategies. Rather than assuming a generalized direct enhancement effect, the results clarify how specific regulatory processes function as mediators within this framework.

From a practical perspective, these findings may inform vocational education settings by suggesting that aesthetic education initiatives could incorporate reflective and meaning-oriented activities aligned with adaptive regulation strategies. However, given the cross-sectional nature of the data, such implications should be interpreted cautiously as practice-relevant hypotheses rather than causal prescriptions.

Because the present sample consisted of Chinese vocational college students, cultural context should be considered when interpreting these findings. Although cognitive reappraisal and expressive suppression are widely studied across cultures, the social meaning and adaptiveness of these strategies may vary depending on cultural norms regarding emotional expression. Future research should examine whether the measurement structure and structural paths identified here are invariant across cultural contexts through multi-group SEM and cross-cultural comparisons.

### 5.2 Limitations

Several limitations of the present study should be acknowledged. First, the cross-sectional design and reliance on self-report measures preclude causal inference. The observed associations are consistent with the proposed mediation model, but reverse or reciprocal relations (e.g., more resilient students being more likely to engage in aesthetic education) and unmeasured third variables cannot be ruled out. Future research employing longitudinal, experimental, or multi-source designs may help to further examine the proposed causal pathways.

Second, all variables were assessed using self-report measures, which may be subject to social desirability bias or common method variance. Although the hypothesized multi-factor measurement model demonstrated substantially better fit than alternative and single-factor models, suggesting that a single common factor does not adequately account for the covariance structure, common method variance cannot be fully excluded. Incorporating time-separated measurement, multi-informant data, or marker-variable approaches in future studies could enhance the robustness of the findings.

Finally, aesthetic education participation was measured without differentiating among specific types or levels of engagement. Whether different forms of aesthetic education operate through similar or distinct psychological mechanisms remains an open question for future research.

In addition, it is important to clarify that “emotion regulation” in the present study was operationalized specifically through two ERQ strategy dimensions—cognitive reappraisal and expressive suppression. These constructs capture important but not exhaustive components of emotion regulation. Future studies should incorporate a broader range of regulatory processes to examine whether the present mechanism generalizes beyond the ERQ framework.

The strength of the proposed mechanism may also vary across individuals and contexts (e.g., student characteristics, disciplinary backgrounds, or institutional climates). Future research could test such boundary conditions via moderation analyses and cross-context replication to refine the generalizability of the model. In addition, quasi-experimental designs—such as comparing cohorts before and after the implementation of structured aesthetic education programs, or using matched comparison groups across institutions—could provide stronger evidence regarding the robustness of the proposed mechanism. Intervention-based studies that integrate aesthetic education modules with explicit emotion regulation training may further clarify whether the observed associations reflect potentially causal processes.

## 6 Conclusion

This study examined the relationships among aesthetic education participation, emotion regulation strategies, and psychological resilience among vocational college students within a theoretically grounded structural model. The findings indicate that aesthetic education participation is associated with psychological resilience primarily through two complementary emotion-regulation pathways—greater cognitive reappraisal and lower expressive suppression. Consistent with the cross-sectional design, these results should be interpreted as evidence of associations rather than causal effects.

At the theoretical level, the study integrates aesthetic education research with emotion regulation and resilience frameworks, extending educational psychology by specifying a mechanism-based account of how educational experiences relate to students' adaptive functioning. By linking aesthetic education participation to resilience through strategy-level regulation processes, the findings underscore the relevance of aesthetic education to broader educational psychology concerns and may inform policy discussions on strengthening psychosocial support within vocational education systems. Future research could employ longitudinal or experimental designs and incorporate multi-source data to further examine causal relationships and deepen understanding of the psychological functions of aesthetic education.

## Data Availability

The raw data supporting the conclusions of this article will be made available by the authors, without undue reservation.
