# Supplementary material for: How aesthetic education enhances psychological resilience: the mediating role of emotion regulation
Source: Front Psychol. 2026 Apr 15;17:1802363. doi: 10.3389/fpsyg.2026.1802363 (PMC13133678; doi:10.3389/fpsyg.2026.1802363)
Supplement: Supplementary file 1 [file Supplementary_file_1.pdf]

Supplementary Table S1: The Questionnaire (Chinese version)

亲爱的同学：

- 本问卷旨在了解职业院校学生的课程体验与心理发展状况。问卷采用匿名方式，所有数据仅用于学术研究，不涉及任何个人评价。请根据您的真实感受作答，答案无对错之分。
- 本研究已通过学术伦理审查，您的参与完全自愿，您可在任何时间退出问卷。
- 感谢您的支持！

[量表说明]

1 = 非常不同意

2 = 不同意

3 = 一般

4 = 同意

5 = 非常同意

A.美育参与（Aesthetic Education Participation）

1. 在校期间，我会定期参与美育相关课程或活动（如艺术课程、艺术实践、审美类课程等）。

2. 在美育课程或活动中，我通常会投入较多的情感和思考。

3. 美育课程为我提供了表达个人情感或想法的机会。

4. 参与美育课程时，我会对自身的感受进行反思和理解。

5. 我认为美育课程对我的个人成长具有积极意义。

B.情绪调节（Emotion Regulation Questionnaire）

（一）认知重评（Cognitive Reappraisal）

6. 当我遇到让我情绪不佳的事情时，我会尝试从不同角度去理解它。

7. 我会通过改变对事情的看法来调节自己的情绪。

8. 当我想减少负面情绪时，我会重新思考当前情境的意义。

9. 面对压力情境时，我会尝试用更积极或理性的方式看待问题。

10. 我能够通过调整想法来改变自己的情绪感受。

11. 即使事情本身没有改变，我也可以通过改变想法让自己感觉好一些。

（二）表达抑制（Expressive Suppression）

12. 我通常会控制自己，不把真实的情绪表现出来。

13. 当我感到不开心时，我倾向于把情绪藏在心里。

14. 即使情绪强烈，我也会尽量不表现出来。

15. 我会压抑情绪表达，以避免被他人察觉。

C.心理韧性（Psychological Resilience）

16. 面对变化时，我能够较好地适应。

17. 即使遇到困难，我也能继续向前。

18. 遭遇挫折后，我通常能够较快恢复过来。

19. 我能够应对生活中的各种压力。

20. 面对挑战时，我会尽力寻找解决办法。

21. 我认为自己是一个有韧性的人。

22. 即使事情进展不顺利，我也不会轻易放弃。

23. 在压力下，我仍能保持一定的冷静。

24. 我相信自己有能力克服困难。

25. 经历困难后，我往往会变得更坚强。

D.人口学信息（Personnel Information）

26. 性别

○ （1）男

○ （2）女

27. 年级

○ （1）一年级

○ （2）二年级

○ （3）三年级

○ （4）其他

28. 年龄

您的年龄是：____ 岁

29. 您所就读的专业类别是：

○ （1）工科 / 技术类（如机电、数控、汽车等）

○ （2）服务类（如酒店管理、旅游、护理等）

○ （3）艺术设计类（如视觉设计、数字媒体、表演等）

○ （4）管理 / 商贸类（如物流、市场营销等）

○ （5）信息技术类（如计算机、软件、人工智能等）

○ （6）其他

30. 您是否修读过本专业以外开设的美育或艺术类课程？

○ （1）是

○ （2）否

31. 您在校期间参与美育课程或活动的大致年限为：

○ （1）少于 1 年

○ （2）1－2 年

○ （3）2 年以上

32. 除学校课程外，您是否参与过课外艺术或审美相关活动（如社团、比赛、兴趣班等）？

○ （1）从未

○ （2）偶尔

○ （3）经常

33. 在进入本校之前，您是否接受过系统性的艺术或美育相关学习？

○ （1）是

○ （2）否

Supplementary Table S2：Skewness and Kurtosis of Observed Variables

Construct	Item	Skewness	Kurtosis
Aesthetic Education Participation（AEP）	AEP1	−0.096	−0.704
	AEP2	−0.220	−0.593
	AEP3	−0.354	−0.252
	AEP4	−0.239	−0.441
	AEP5	−0.202	−0.599
Aesthetic Education Participation（CR）	CR1	−0.160	−0.454
	CR2	−0.219	−0.724
	CR3	−0.224	−0.603
	CR4	−0.138	−0.797
	CR5	−0.213	−0.667
	CR6	−0.184	−0.679
Expressive Suppression（ES）	ES1	−0.056	−0.374
	ES2	0.105	−0.456
	ES3	−0.052	−0.470
	ES4	0.156	−0.320
Psychological Resilience（PR）	PR1	−0.343	−0.377
	PR2	−0.302	−0.544
	PR3	−0.268	−0.510
	PR4	−0.309	−0.465
	PR5	−0.369	−0.516
	PR6	−0.336	−0.465
	PR7	−0.308	−0.563
	PR8	−0.246	−0.268
	PR9	−0.329	−0.501
	PR10	−0.309	−0.494

Note.Skewness values ranged from −0.37 to 0.16 and kurtosis values ranged from −0.80 to −0.25. All values fell within commonly accepted thresholds (|skewness| < 2; |kurtosis| < 7), indicating no substantial deviations from normality. No extreme outliers were identified based on descriptive inspection.

Supplementary Table S3: Standardized Factor Loadings for the Measurement Model

Item			Standardized Loading
AEP1	<---	AEP_lat	0.707
AEP2	<---	AEP_lat	0.735
AEP3	<---	AEP_lat	0.723
AEP4	<---	AEP_lat	0.715
AEP5	<---	AEP_lat	0.677
CR1	<---	CR_lat	0.659
CR2	<---	CR_lat	0.613
CR3	<---	CR_lat	0.646
CR4	<---	CR_lat	0.654
CR5	<---	CR_lat	0.670
CR6	<---	CR_lat	0.638
ES1	<---	ES_lat	0.668
ES2	<---	ES_lat	0.702
ES3	<---	ES_lat	0.655
ES4	<---	ES_lat	0.709
PR1	<---	PR_lat	0.642
PR2	<---	PR_lat	0.663
PR3	<---	PR_lat	0.669
PR4	<---	PR_lat	0.674
PR5	<---	PR_lat	0.676
PR6	<---	PR_lat	0.668
PR7	<---	PR_lat	0.638
PR8	<---	PR_lat	0.641
PR9	<---	PR_lat	0.590
PR10	<---	PR_lat	0.662
